# Editorial: New challenges and trends in rehabilitation devices based on AI and optimization

**DOI:** 10.3389/frobt.2023.1248973

**Published:** 2023-09-08

**Authors:** Pedro Ponce, Mariel Alfaro-Ponce, Edgar Omar López-Caudana, Troy McDaniel, Luis Montesinos, Jesús Ricardo López-Gutiérrez, Esther Lugo-González

**Affiliations:** ^1^ School of Engineering and Science, Tecnologico de Monterrey, Mexico City, Mexico; ^2^ Arizona State University, Tempe, AZ, United States; ^3^ Investigador por México-Consejo Nacional de Humanidades, Ciencias y Tegnologías (IXM-CONAHCYT), Mexico City, Mexico; ^4^ Institute of Electronics and Mechatronics, Universidad Tecnológica de la Mixteca, Oaxaca, Mexico

**Keywords:** artificial intelligence, robots, theraphy, computer vision, machine learning (ML)

This editorial paper presents a comprehensive overview of recent advancements and breakthroughs in artificial intelligence (AI) and neural networks, with a particular focus on their applications in various fields such as human activity recognition, medical image steganography, lower limb prosthetics, assistive robots for Autism Spectrum Disorder (ASD), and wearable assistive devices for visually impaired individuals. The paper discusses notable developments in spiking neural P systems, genetic-based steganography algorithms, systematic analysis of prosthetic technology, energy optimization of autonomous assistive robots, and the Tiny-Yolov3 architecture for pedestrian detection. AI algorithms employed for human activity recognition often surpass the computational capabilities of current computing systems due to factors such as camera movement, complex scenes, and occlusions. To address this problem, researchers and practitioners have proposed a solution based on selective visual attention, a process inspired by human visual perception that has increased its potential. Recent studies have introduced a spiking neural P system, which efficiently extracts features from human motion, significantly enhancing the performance of neural classifiers with low computational complexity in human action recognition (Anides et al.); thus, they can be used for being deployed into embedded digital systems. Steganography is crucial in safeguarding information in advanced applications like medical image communication and personal data storage (Vazquez et al.). However, most existing steganography approaches tend to introduce some distortion in the cover image, which can be detected through steganalysis techniques. To mitigate this disadvantage, recent work suggests utilizing a genetic algorithm-based steganography method that minimizes image distortion, thereby improving the Peak Signal to Noise Ratio (PSNR) levels. Furthermore, there have been substantial advancements in powered prostheses, greatly enhancing the quality of life for individuals with lower limb disabilities (Dominguez-Ruiz et al.). These technological developments have yielded significant improvements in lower limb prosthetics. Wearable assistive devices have been developed to aid visually impaired individuals in their day-to-day activities. These devices utilize AI and sensor technologies to provide real-time feedback and assistance, empowering visually impaired individuals to navigate their surroundings more effectively (Maya-Martínez et al.). As a result, they can recover mobility and freedom in a high percentage. In addition, assistive robots designed specifically for individuals with autism spectrum disorder (ASD) have emerged as a promising area of research (Fuentes-Alvarez et al.). These robots are tailored to support and assist individuals on the autism spectrum, helping them improve their social skills and overall wellbeing. Nowadays, they are crucial tools for therapy that can improve social skills in a short period.

In conclusion, this editorial paper highlights the remarkable progress made in AI and neural networks, shedding light on their applications in human activity recognition, medical image steganography, lower limb prosthetics, assistive robots for ASD, and wearable assistive devices for visually impaired individuals. The discussed advancements in spiking neural P systems, genetic-based steganography algorithms, systematic analysis of prosthetic technology, energy optimization of autonomous assistive robots, and the Tiny-Yolov3 architecture demonstrate the transformative potential of AI in improving various aspects of human life. A systematic literature review of the current technologies for developing lower limb prosthetics reveals a need for a specific, generalized structure for new developments. Notable gaps exist in energy management and improve, smoother patient interaction, thereby highlighting areas for future research and improvement. The application of socially assistive robots for autism therapies is increasing. A recent study presents the integration of an output feedback adaptive controller for trajectory tracking and energetic autonomy of a mobile socially assistive robot for ASD under an event-driven control scheme. Energy optimization is crucial as it allows therapists to extend and adapt sessions with autistic children, enhancing effectiveness during therapies. Finally, wearable assistive devices based on video camera technology pose unique challenges for visually impaired individuals. A proposed Tiny You Only Look Once (YOLO) architecture for pedestrian detection can be implemented in low-cost wearable devices, providing a potential solution for assistive technologies for the visually impaired. In conclusion, advancements in artificial intelligence and neural networks continue to revolutionize numerous fields, improving both the efficiency and quality of solutions. However, continued research and improvements are necessary to harness these technological breakthroughs and optimize their benefits. These five research papers illuminate the cutting-edge advancements and future potentials of artificial intelligence (AI) and neural networks in various fields, ranging from human activity recognition to medical image confidentiality, prosthetics, assistive robots for autism spectrum disorder (ASD), and wearable devices for the visually impaired. Each paper individually contributes to the broader landscape of AI, offering innovative solutions to existing challenges and opening new avenues for exploration. They reveal the transformative power of AI in enhancing real-time applications, increasing computational efficiency, improving user interactions, ensuring data confidentiality, and optimizing energy management. Yet, collectively, they tell a more profound story of how interconnected these AI applications are and how common threads weave through them. They reinforce the notion that the evolution of AI is not happening in silos but is a holistic, intertwined process where advancements in one area can catalyze growth in others. As AI continues to develop, researchers, developers, and practitioners must consider this interconnectedness. This approach will allow us to fully realize the potential of AI, not only to solve individual problems but also to create systemic changes that ripple across sectors and applications. AI is not only a tool or a participant in the process but a fundamental shift in how we perceive, interact with, and shape our world. The progress discussed in these papers is inspiring, yet it is equally a reminder of the endless journey ahead in AI research. The opportunities for AI to further revolutionize healthcare, accessibility, and our daily interactions are vast, and our collective pursuit of these advancements is what will continue to drive progress in this exciting, transformative field.

